# Experimental Designs for Preclinical Neuroscience Experiments: Part 2—Blocking and Blocked Designs

**DOI:** 10.1523/ENEURO.0006-26.2026

**Published:** 2026-02-24

**Authors:** P. S. Reynolds

**Affiliations:** Department of Anesthesiology, College of Medicine, University of Florida, Gainesville, Florida 32610

**Keywords:** blocking, design of experiments, incomplete block, Latin square, randomized complete block, statistics

## Abstract

Blocking is a key statistical method introduced almost a century ago by Ronald Fisher. Blocking controls the effect of “nuisance” variables that are not of direct interest but introduce unwanted variation into the experimental response. Block factors, such as cage, litter, or time, are used to group experimental units into homogeneous subsets. There are two types of block designs: complete and incomplete. In complete block designs every treatment appears in every block. Examples include the Randomized Complete Block Design (RCBD) with a single block factor, and variants such as Latin square and Graeco-Latin square designs with multiple block factors. RCBDs are simple, flexible, and the most widely used. Replicated and nested Latin square designs allow more rigorous control of complex nuisance structures with minimal sample size. Incomplete block designs are extremely useful when practical constraints (e.g., caging density or varying litter sizes) restrict complete treatment replication across all blocks. Because not all treatments appear in every block, these designs require computer-generated allocation plans to obtain optimum balance and efficiency. Each of the seven blocking designs described in this paper includes a practical example from the research literature, the corresponding skeleton analysis of variance and R code for random allocation plans. By increasing precision and power to detect treatment effects, blocking promotes ethical research by maximizing the amount of information for a minimum number of animals, supporting the 3Rs principle of Reduction.

## Introduction

Blocking is one of the most important contributions made by Sir Ronald Fisher to the statistics of experimental design (Box, 1980). By systematically controlling unwanted variation resulting from the so-called “nuisance” variables, blocking can increase the strength of the experimental signal and increase the precision and reliability of results. In the groundbreaking book *Principles of Humane Experimental Technique*, Russell and Burch (1959) suggested that designed experiments that could isolate and control as many sources of variation as possible were a key tool for increasing the information of an experiment while minimizing the number of animals needed for experiments.

In this paper, blocking strategies are presented for seven commonly encountered experimental situations. A skeleton ANOVA is shown for each design, to show how degrees of freedom are partitioned among the different sources of variation, enabling the correct setup of the analysis of variance model and a check on the validity of analysis outputs. R code is provided in Extended Data 1.

## What Is Blocking?

Blocking is a statistical design method used to control and reduce unwanted variation between experimental units. Blocks are groups of experimental units where members within each block are as similar as possible. Treatments are then randomly allocated to the experimental units within each block.

The block (or nuisance) factor is a categorical classifier usually external to the subject. Unlike explanatory or predictor factors, the block factor is not of direct interest to the research question, but it can affect the response indirectly by increasing unwanted variation and obscuring the treatment effect signal. Including the block factor in the analysis accounts for the variation associated with the nuisance variable and removes it from the error variance. As a result, the error variance is reduced and the treatment *F* statistic increases, increasing the power to detect treatment differences. In spite of the cost of a few degrees of freedom associated with the block factor, blocked designs are more powerful than completely randomized designs (Cochran and Cox, 1957).

The block factor can be a physical grouping variable such as cage, litter, pen, tank, or donor. For example, animals housed together in the same cage share a common physical and social environment and therefore are more similar to each other than to animals in other cages. Blocking by cage is therefore a sensible strategy. Block factors can also be defined by management criteria, such as different labs, personnel, and time periods (such as day or week). Management-based block factors can provide excellent control of variation resulting from protocol disruptions and performance differences in technical staff, as well as unknown and unplanned time-based environmental fluctuations. Suppose an experiment takes 4 d to run. It is possible that small daily changes or fluctuations in the operating environment could occur that would contribute to variation in the results. One option for this experiment would be to block by day, with one-quarter of the units processed on each day. Any time differences between days are accounted for by differences between blocks.

In blocked designs replicates refer to the number of blocks of each complete set of treatments. Therefore, sample size calculations for a blocked design require an estimate of the number of blocks (not “group size”) required to achieve a desired power (Steel and Torrie, 1980). For example, sample size for a randomized complete block design (RCBD) is the number of blocks *b* needed to detect the predetermined treatment effect size for a given power (1 − *β*) and significance level (*α*). The total sample size *N* is *b*·*t*, where *t* is the number of treatments. Rigorous sample size calculations may require iterative estimates based on the noncentrality parameter (Reynolds, 2024). However, reasonable first-pass approximations may be obtained by constructing a skeleton ANOVA and computing error degrees of freedom with a range of block sizes: Mead (1988) recommends a sample size that allows 10–20 degrees of freedom for the error term.

**Figure 1.**
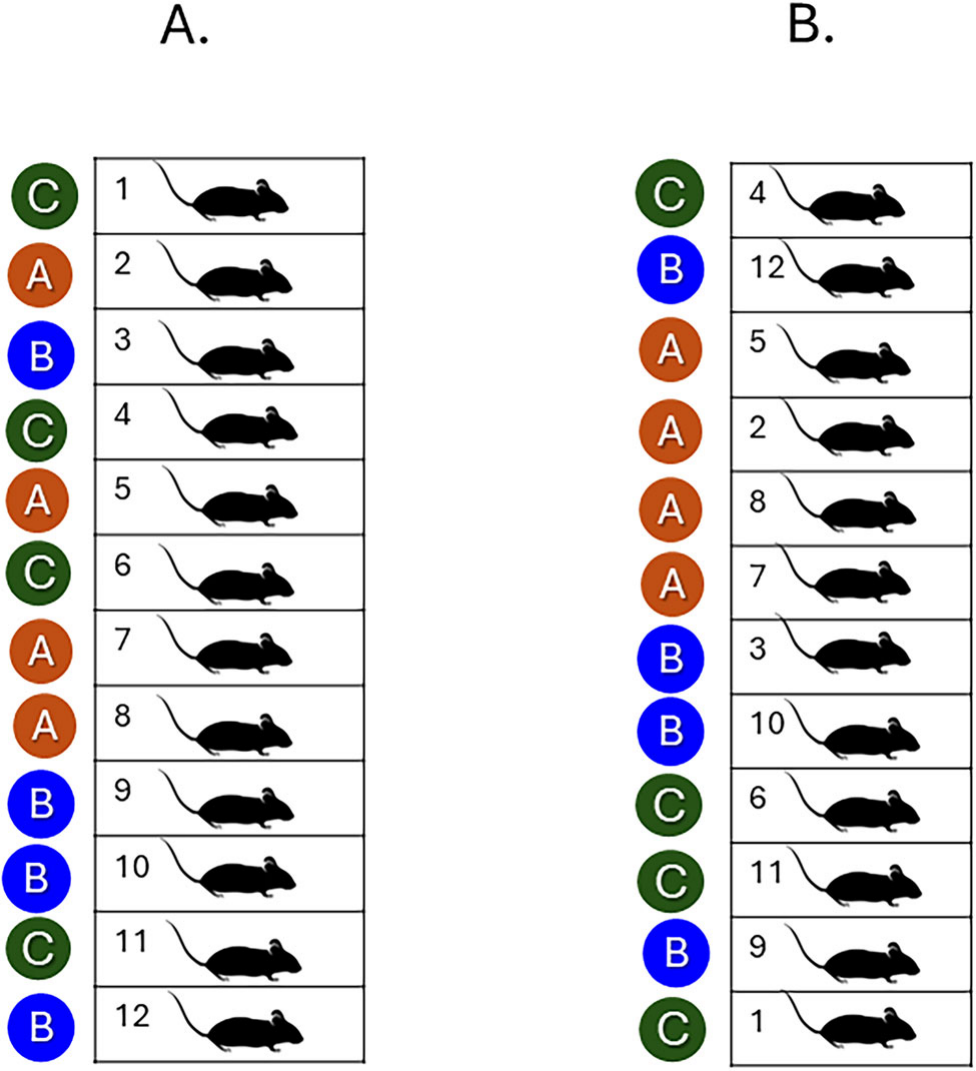
Random treatment allocation and random sequence allocation. Randomization minimizes bias and is essential for reproducibility and rigor. ***A***, Random treatment allocation: Treatments are randomly allocated to the experimental units. ***B***, Random sequence allocation. During data collection, order of processing is randomized.

After the number of experimental units and block structure have been determined, the allocation of treatments to the experimental units is randomized according to a design-specific plan (random treatment allocation). Randomization treatment allocation plans must be tailored to the specific design. If measurements are obtained sequentially during the data collection phase, it is also advisable to randomize processing order (random sequence allocation; Fig. 1, Extended Data Fig. 1-1). Random sequence allocation shuffles the order of the experimental unit identifiers and can usually be performed by simple randomization algorithm without replacement. Both random treatment and sequence allocation plans should be generated with validated randomization methods. Appropriate randomization is recommended by the ARRIVE 2.0 guidelines as essential to best practice experimentation (Percie du Sert et al., 2020).

10.1523/ENEURO.0006-26.2026.d1 Data 1 Download Data 1, DOCX file.

Appropriate randomization procedures require experimental units to be individually identifiable. For example, if individual mice within a cage are the experimental units, the mice should be individually identified by, e.g., ear-tags, RFID microchips, or indelible sharpie marks on the tail.

## Types of Blocked Designs

There are two major categories of blocked designs: complete block and incomplete block.

### Complete block designs

In complete block designs, all treatments are represented in each block, all blocks are equal in size, and block size is equal to, or is a multiple of, the number of treatments. The basic blocked design is the randomized complete block design (RCBD) with one block factor. More complex block designs include Latin square designs with two blocking factors and Graeco-Latin square designs with three blocking factors.

#### Randomized complete block design

**Figure 2.**
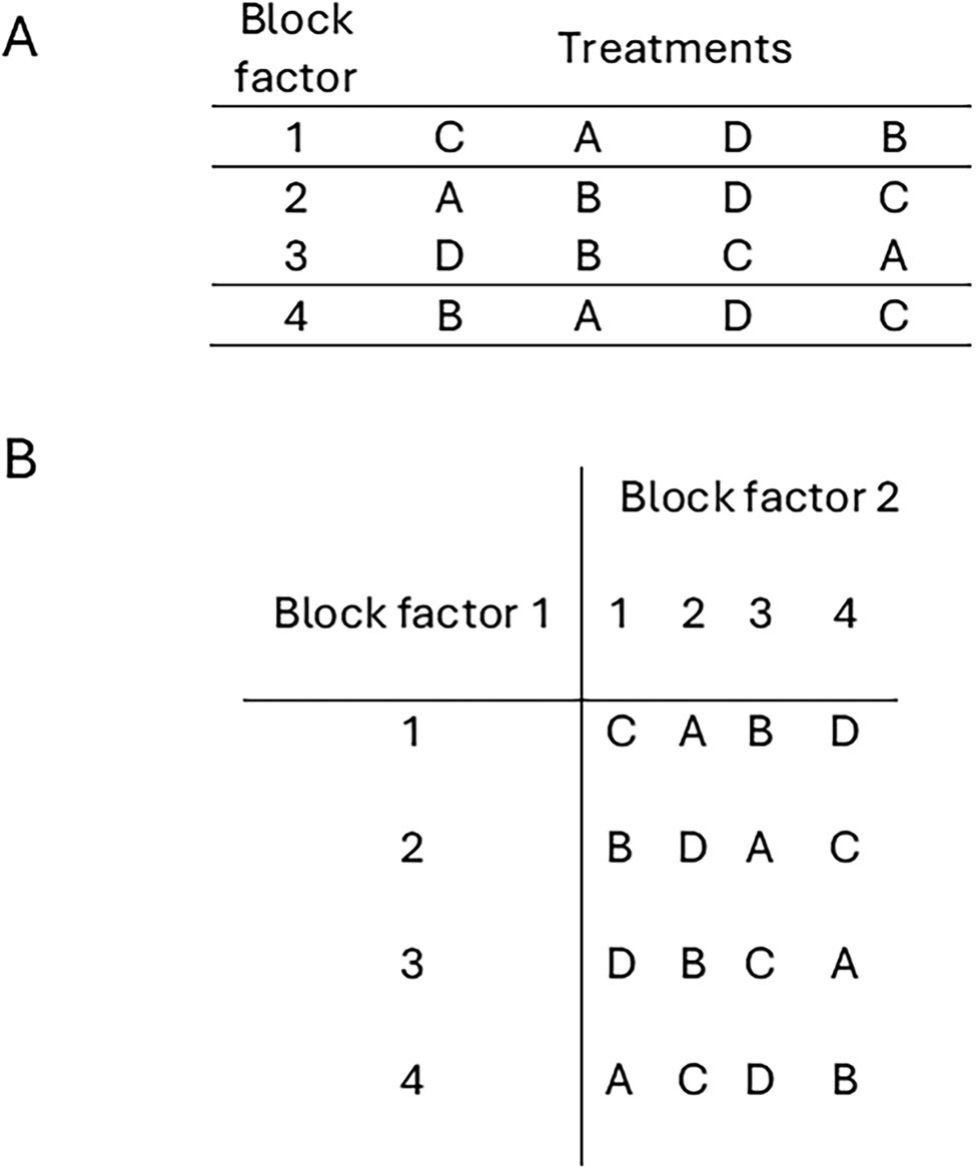
Sample random allocation plan for randomized complete block and Latin square designs, each with four treatments (A, B, C, D). ***A***, Randomized complete block design with one block factor. Treatments are randomly allocated to the experimental units within each block. ***B***, 4 × 4 Latin square design on two blocking factors. Each treatment occurs only once in each row and each column.

The randomized complete block (RCB) design is the workhorse of blocked designs. It is simple to construct and analyze, flexible (because it can accommodate any number of treatments and blocks), and robust (because one or two missing data points do not invalidate the analysis). The RCBD is therefore readily applicable to a variety of experimental situations. The RCBD design is balanced; that is, each block contains all the treatments, and each treatment occurs the same number of times in each block. Treatments are randomly assigned to the experimental units in each block, so that each treatment appears at least once in each block (Fig. 2; Extended Data Fig. 2-1).

10.1523/ENEURO.0006-26.2026.d2 Data 2 Download Data 2, DOCX file.

A limitation of RCB designs is that only one block factor at a time can be considered. If more than one nuisance variable might be important, the researcher should consider Latin square or other, more complex, blocked designs.

*Example*. A researcher planned to investigate the effect of three drugs (B, C, D) compared with a vehicle control (A) on brain oxygen concentration (BrPO_2_) of 24 mice housed four to a cage. The factor is Drug with four levels (A, B, C, D) and therefore there are *t* = 4 treatments. The response variable is BrPO_2_. The experimental unit is the individual mouse and the block factor *b* is the cage. The four treatments must be randomly allocated to the four mice in each cage, so all treatments appear in each cage.

To demonstrate the utility of blocking when unwanted variation is large, the BrPO_2_ data (Extended Data 2, Table 1-1) were analyzed with and without the block factor (Table 1). If the effect of cage is ignored in the analysis, differences between drugs are not statistically significant. Incorporating the block factor removes the variation associated with cage effects from the error variance, and the drug effect is now flagged as highly significant. Because blocking allows comparison of similar experimental units to each other, any differences in outcomes are more likely to be attributable to treatment effects rather as artefact resulting from pre-existing differences between the experimental units.

**Table 1.** Blocking increases efficiency of analysis of variance

Source of variation	df	Sum of squares	Mean square	*F*	*p* value
Block factor omitted					
Treatment = drug	*t* − 1 = 3	11.22	3.74	0.59	0.63
Error (residual)	*t*(*n* − 1) = 20	126.44	6.32		
Block factor included					
Treatment = drug	*t* − 1 = 3	11.22	3.74	9.89	0.0008
Block = cage	*b* − 1 = 5	120.76	24.15	63.82	<0.0001
Error (residual)	(*t* − 1) (*b* − 1) = 15	5.68	0.38		

Comparison of the sum of squares terms shows that the block factor accounts for most of the variation in the error term. Removing the unwanted variation associated with between-cage variation strengthens the treatment signal.

#### Latin square designs

In a Latin square design, there are two block factors and one treatment factor, all of which must have the same number of levels. That is the number of levels for both blocking factors must match the number of treatment levels. The Latin square design balances the treatments across both block factors at the same time. There is one observation per treatment represented in each row and column. The random treatment allocation plan is displayed as a grid, with block factors as rows and columns, and treatments randomly allocated to cells within the grid (Fig. 2; Extended Data 1, 2-2). Randomization for a Latin square design consists of random permutation of the columns, then rows, followed by random allocation of treatments to each cell. If there are two or more replicates, randomization is performed separately for each replicate.

The structure of the Latin square ANOVA model depends on whether the design is replicated and if the levels of one or both block factors are the same across all replicates. Latin square designs also assume that there are no interactions either between block factors, and no interaction between block factors and treatments. It is important to clarify the appropriate model structure before the experiment is conducted, because the wrong model structure will result in incorrect formulation of the error degrees of freedom.

*Unreplicated Latin square designs*. Unreplicated Latin square designs are common in studies when time is used for one or both blocking factors, and there neither time nor resources to permit additional replicates. Because there are no replicates, the researcher must check that there are sufficient degrees of freedom for the error variance. There is also the risk that true differences between treatments may not be detected because of the small sample size. Unreplicated Latin square designs are probably best for preliminary pilot investigations or screening experiments rather than definitive confirmatory experiments. Blocking on a time dimension such as day and week effectively creates a repeated-measures design if all treatments are represented across the different time points and the same subjects are measured repeatedly. However, the effects of time are not tested directly; instead, blocking on time balances out any time trends.

*Example*. The effects of distinct types of music as a form of enrichment have been studied in cattle and other livestock (Ciborowska et al., 2021; Pinkerton et al., 2025) and laboratory rodents (Kühlmann et al., 2018). The logistics of allocating auditory treatments could be extremely challenging. However, a Latin square design with blocking on both day and week would be relatively simple to execute, because each auditory treatment could be randomly allocated to the entire housing unit by day and by week. Because the entire housing unit is the experimental unit, and the daily response is averaged, the number of animals or cages within the housing unit is not a critical part of the analysis.

An experiment was designed to assess four types of music (classical A, rock B, jazz C, country D) against a no-music control (E) on well-being of a colony of laboratory mice. It was hypothesized that increased benefit would be demonstrated by an increase in average daily activity. The music type allocated for a given day was played over the entire housing unit. The experiment was to be run 5 d per week (Day, block factor 1) over 5 weeks (Week, block factor 2). The observational unit is the housing colony. The response is daily activity averaged over all mice in the colony.

**Figure 3.**
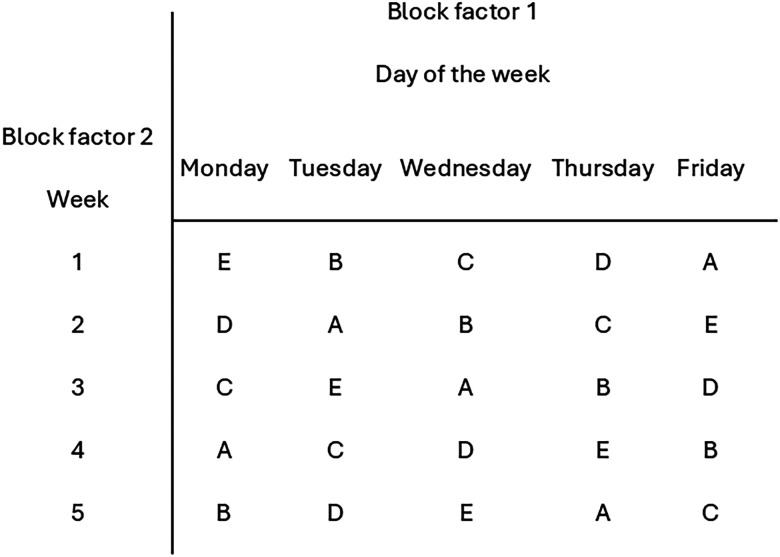
Unreplicated 5 × 5 Latin square design for a study of the effect of music on average daily activity of laboratory mice. The Music treatment has five levels (classical A, rock B, jazz C, country D, no-music control E) blocked by Day of the week (block factor 1) and Week (block factor 2).

Figure 3 shows a sample allocation plan for the 5 × 5 Latin square design. It is seen in the accompanying skeleton ANOVA (Table 2) that the error term has 12 degrees of freedom, which is sufficient for testing the effect of treatment.

**Table 2.** Skeleton ANOVA for an experiment testing the effects of music on average daily activity of a mouse colony

Source of variation		Degrees of freedom	
Treatments	Type of music	*t* − 1	5 − 1 = 4
Row block factor	Week	*t* − 1	5 − 1 = 4
Column block factor	Day	*t* − 1	5 − 1 = 4
Error		(*t* − 1) (*t* − 2)	(5 − 1) (5 − 2) = 12
Total		*t*^2^ − 1	5_·_5 − 1 = 24

There were five treatments (classical music A, rock B, jazz C, country D, no-music control E). Treatments were allocated as a 5 × 5 Latin square design on the block factors Day and Week, and daily activity measurements were averaged over all mice in the colony.

*Replicated Latin square designs*. Usually, the researcher will wish to replicate observations as these increase the robustness of an experiment. There are three basic forms of the Latin square ANOVA for a replicated experiment, depending on how the block factors are structured across the replicates: no nesting of block levels, nesting of one block factor within replicates, or nesting of both block factors within replicates. The model structure that will be most appropriate for the research problem must be decided before the experiment is conducted, as the wrong model will result in incorrect formulation of the error degrees of freedom and invalidate results.

*No nesting of block levels*. This Latin square model is appropriate when both block levels are the same across all replicates; that is, the components of the rows and columns are the same across all replicates.

**Figure 4.**
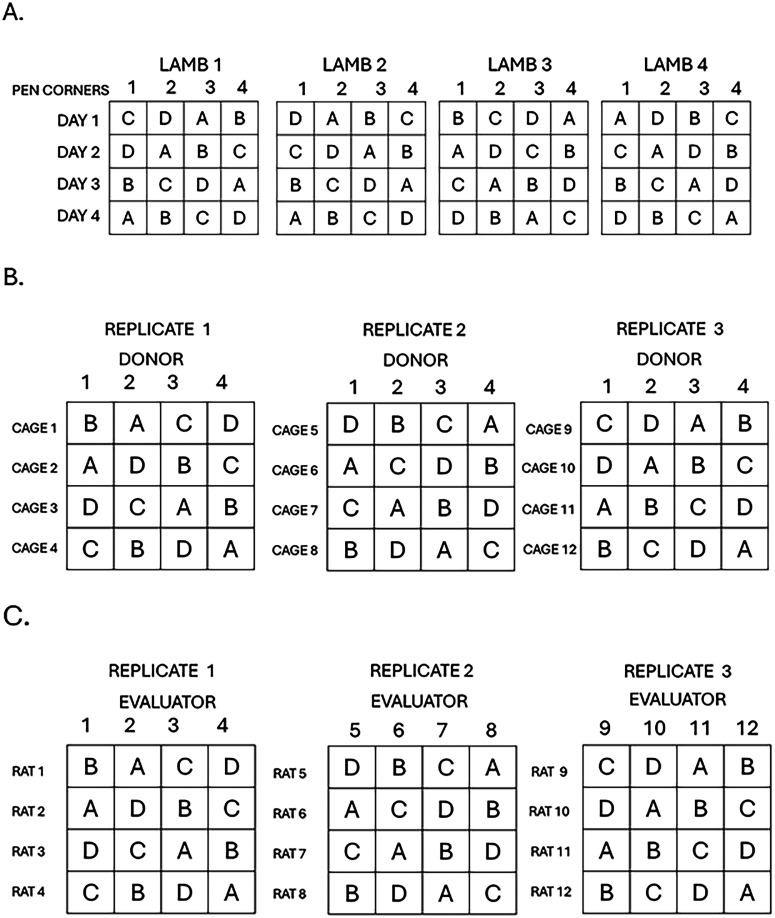
Replicated Latin square designs. ***A***, No nesting, block levels are the same across all replicates: Lamb feeding study. ***B***, One nested blocking factor: Tumor inhibition in a PDX mouse model. ***C***, Two nested block factors: Rat brain imaging study.

*Example*. A dietary preference study was conducted on four lambs (Borman et al., 1991). Each lamb was kept in a separate pen and fed one of four grass types over 4 d. Fodder was placed in a different randomly selected corner of the pen each day to minimize potential positioning bias by the lambs. Day (block factor 1) and pen corner (block factor 2) are the same across the four lambs (the replicates). The design is randomized separately for each lamb (Fig. 4*A*). With *t* = 4 treatments replicated across *r* = 4 lambs, the ANOVA has (*t* − 1) (*rt* − 2) = 30 degrees of freedom for the error (Table 3A; Extended Data 1, 3-1).

**Table 3.** Skeleton ANOVAs for replicated 4 × 4 Latin square designs adjusted for nesting of block factors within replicates

Source of variation		Degrees of freedom	
A. Block levels the same across four replicates, no nesting: Lamb feeding trial			
Replicate	Lamb	*r* − 1	4 − 1 = 3
Treatments	Forage type	*t* − 1	4 − 1 = 3
Row block factor (same)	Day	*r*(*t* − 1)	4(4 − 1) = 12
Column block factor (same)	Pen corner	*t* − 1	4 − 1 = 3
Error		(*t* − 1) (*rt* − 2)	(4 − 1) (4_·_4 − 2) = 42
Total		*rt*^2^ − 1	4_·_4_·_4 − 1 = 63
B. One nested block factor, three replicates. Tumor inhibition in a PDX model			
Replicate	Day	*r* − 1	3 − 1 = 2
Treatments	Drug	*t* − 1	4 − 1 = 3
Row block factor (new)	Cage	*t* − 1	4 − 1 = 3
Column block factor (same)	Donor	*r* (*t* − 1)	3(4 − 1) = 9
Error		(*r* − 1) (*t*^2^ − 1) + (*t* − 1) (*t* − 2)	(3 − 1) (4^2^ − 1) (3) (2) = 36
Total		*rt*^2^ − 1	3_·_4_·_4 − 1 = 47
C. Both block factors nested, three replicates			
Replicate	Laboratory	*r* − 1	3 − 1 = 2
Treatments	Imaging method	*t* − 1	4 − 1 = 3
Row block factor (new)	Rat	*r* (*t* − 1)	3(4 − 1) = 9
Column block factor (new)	Evaluator	*r*(*t* − 1)	3(4 − 1) = 9
Error		(*r* − 1) (*t*^2^ − 1) − (*t* − 1) (*t* − 2)	(2) (15) − (3) (2) = 24
Total		*rt*^2^ − 1	(3)(4)(4) − 1 = 47

*One nested blocking factor*. In this scenario, components of one blocking factor will be the same across all replicates but will be new for the second block factor. The block factor with unique components will be nested within replicate and this nesting must be incorporated in the ANOVA model.

*Example*. A researcher plans to test four drugs A, B, C, D (treatments) for their effects on tumor inhibition in a patient-derived xenograft (PDX) mouse model. Tumor tissue from four donors will be transplanted into immunodeficient mice. Recipient mice are caged in groups of four and are individually identifiable. Donor and Cage are considered block factors. They are not of direct interest to testing the hypothesis of drug effect but could contribute unwanted variation to measurements of the response of individual mice. Because the number of donors is limited, and tissue from any one donor can be allocated to multiple mice, the most efficient design will have the same donors represented across all replicates. Donor is block factor 1, and block levels represent individual donors. Cage is block factor 2. Each mouse in each cage receives a tumor implant from one donor, so all four donor sources are represented in each cage. The experiment is to be replicated over 3 d with different cages of mice implanted on each day. With three replicates, each donor contributes tissue to 12 recipient mice, so there are three mice for each donor and treatment combination (Fig. 4*B*). Cages of mice used for each replicate are unique, so the block factor for cage is nested within day. With this design, the ANOVA has 36 degrees of freedom for the error (Table 3B; Extended Data 1, 3-2).

*Both blocking factors nested within replicates*. In this scenario, the components for both the rows and columns are new for each replicate, so both are nested within replicates and two nested terms must be included in the ANOVA model.

*Example*. A researcher wishes to evaluate estimates of rat brain tumor volumes obtained from three imaging techniques and a histological control image (treatments). All four image types were obtained for each rat. Twelve evaluators from three different laboratories are to be presented with 48 images from a total of 12 rats (4 rats per laboratory). The goal is to assess the effects of the imaging method on the reliability of the brain volume estimates obtained for each imaging method. The researcher is not interested in differences between rats or evaluators but is concerned about unwanted variability. The design was a 4 × 4 Latin square design, with four treatments (imaging method), two block factors (rat, evaluator), and three replicates (laboratories). Because the individual rats and evaluators are unique to each replicate, both block factors are nested in each replicate (Fig. 4*C*). The ANOVA has (*t* − 1)[*r*(*t* − 1) − 1] = 24 degrees of freedom for the error (Table 3C; Extended Data 1, 3-3).

### Incomplete block designs

Many situations arise in experiments when the number of treatments does not match the number of experimental units available for a block. This will make it difficult to balance treatments across the design. Suppose the researcher wishes to block on cage, with the individual mouse in each cage as the experimental mice. However, mice are commonly housed five per cage. If all mice are to be used, five treatments would be required for a complete block design. An experiment with three treatments could house three mice per cage. However, reducing cage density is not always desirable because more cages and shelf space will be required and per diem costs will increase. At the other extreme, the size of natural block factors such as litter or dam may differ a lot between the individual block units. For example, a researcher may wish to control for litter effects by using litter as a block factor with individual pups as experimental units. However, litter size in some strains of mice can vary considerably between dams. The usual solution is to balance the design by culling extra pups, which is wasteful and potentially inhumane.

Incomplete block designs can accommodate block imbalances caused by logistic constraints. Block imbalances occur when the number of treatments is larger than the number of experimental units per block, if the block size would be too large in practice to ensure within-block homogeneity, or if certain combinations of treatment or block combinations are not possible. Incomplete block designs differ from complete block designs by how balance is achieved. In complete block designs, balance is achieved by equal representation of all treatments in every block (Cochran and Cox, 1957). In incomplete block designs, not all treatments appear in every block. Instead, balance is achieved by equal representation of each pair of treatments across all blocks (Table 4).

**Table 4.** Distribution of treatments in a randomized complete block design (RCBD) compared with a balanced incomplete block design (BIBD)

Randomized complete block design (RCBD)					Balanced incomplete block design (BIBD)				
Block	Treatment				Block	Treatment			
	A	B	C	D		A	B	C	D
1	1	1	1	1	1	1	1	1	0
2	1	1	1	1	2	1	1	0	1
3	1	1	1	1	3	1	0	1	1
4	1	1	1	1	4	0	1	1	1

Treatment present = 1, treatment absent = 0. Both designs have four treatments (A, B, C, D) and four replicates (blocks). The RCBD has an equal number of treatments across all blocks and block size is 4. The number of treatments within a block for the BICB design is less than the total number of treatments; block size is 3. Balance in this design is achieved by an equal number of treatment pairs across all blocks; in this example each treatment pair is represented twice.

Given these operational constraints, computer-intensive optimization methods are a good option for providing the most efficient designs possible. The “best” design is selected based on some optimality criterion such as maximal efficiency, minimal variance, or prediction precision. The so-called A-efficient designs minimize the average variance of pairwise treatment differences, making for smaller average standard errors for the effect estimates. They are most useful in screening studies where the goal is to identify the most influential subset of explanatory variables from a larger candidate pool. However, they can be unduly influenced by outliers or model misspecification. D-efficient designs minimize the overall or generalized variance of simultaneous treatment comparisons. They are often preferred in practice because they are robust to model misspecification and are computationally faster. The R packages *ibd* and *AlgDesign* are convenient and rapid methods of generating optimal incomplete block designs.

#### How to construct an incomplete block design

An incomplete block design is described by five parameters: the number of treatments (*v*), the number of blocks (*b*), the number of times each treatment appears in the design (replications; *r*), the number of experimental units assigned to each experimental group (*k*), and the number of times each treatment pair appears in the design (*λ*). The total number of experimental units *N is bk* = *rg*. The design must satisfy two conditions for the optimal design to exist: the number of replicates *r* must equal *bk* */* *v*, and the number of treatment pairs *λ* must equal *r*(*k *− 1) / (*v *− 1). The solutions must be integers.

#### Number of treatments exceeds block size

Suppose a researcher is planning an experiment with six treatments (A through F), with cage as a block factor and the individual mouse as the experimental unit. However, there are five mice per cage. In this example, there are *v* = 6 treatments but only *k* = 5 experimental units per block. We first solve for the number of replicates *r* and for blocks *b*:

r=bk/v=b(5)/6.

**Figure 5.**
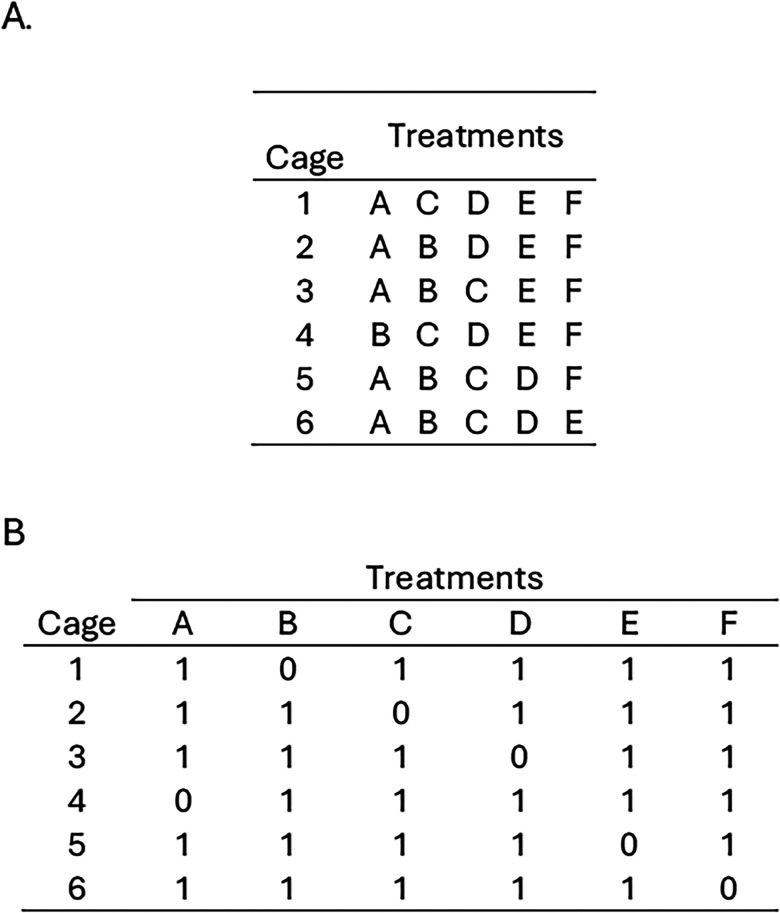
Random allocation plan for a balanced incomplete block design when number of treatments is larger than block size. ***A***, Sample random allocation plan. ***B***, Distribution of treatments (present = 1, absent = 0) showing that treatments are balanced across the entire experiment rather than by block: each treatment is represented five times and each treatment pair four times.

The solution must be an integer, so the fraction is rounded up to the next whole number. The lowest whole number occurs with *b* = 6 cages, so *r* = 5 and *N* = 6 × 5 = 30 mice. Solving for *λ* we obtain *r*(*k *− 1)/(*v* − 1) = 5(5 − 1)/5 = 4 treatment pair replicates. For example, treatments A and C will appear four times in the design (as will every other treatment pair). Once the parameters have been quantified, the design can be generated in R library (*ibd*). The randomization plan allocates the six treatments A through F so that the five treatment replicates and four treatment pair replicates are evenly distributed throughout the experiment (Fig. 5; Extended Data 1, 5-1). In this example, the R command *bibd*(*v,b,r,k, λ*) is entered as *bibd*(6,6,5,5,4).

In the ANOVA, the sum of square for treatments must be adjusted for the block effects because not all treatments appear in all blocks. The error degrees of freedom are calculated by subtracting the degrees of freedom for the blocks and the adjusted sum of squares from the total (Table 5).

**Table 5.** Skeleton ANOVA for three types of incomplete block designs

Source of variation		Degrees of freedom	
A. Block size is smaller than the number of treatments: Six treatments, block factor is cage, 5 mice per cage			
Block	Cage	*b* − 1	6 − 1 = 5
Treatments	A through F	*t* − 1	6 − 1 = 5
Error		(*N* − 1) − (*b* − 1) − (*t* − 1) = *N* − *b* − *t* + 1	(30 − 1) − (6 − 1) − (6 − 1) = 19
Total		*N* − 1	30 − 1 = 29
B. Block size is greater than number of treatments: Three treatments, block factor is cage, 5 mice per cage			
Block	Cage	*b* − 1	6 − 1 = 5
Treatments	A, B, C	*t* − 1	3 − 1 = 2
Error		*N* − *b* − *t* + 1	30 − 6 − 3 + 1 = 22
Total		*N* − 1	30 − 1 = 29
C. Variable block sizes: Three treatments, litter as block factor, litter size varies between 3 and 12			
Block (litters)	Litter	*b* − 1	12 − 1 = 11
Treatments	A, B, C	*t* − 1	3 − 1 = 2
Error		*N* − *b* − *t* + 1	30 − 6 − 3 + 1 = 93
Total		*N* − 1	107 − 1 = 106

A. Block size is smaller than the number of treatments. B. Block size is greater than number of treatments. C. Variable block sizes.

#### Block size exceeds number of treatments

**Figure 6.**
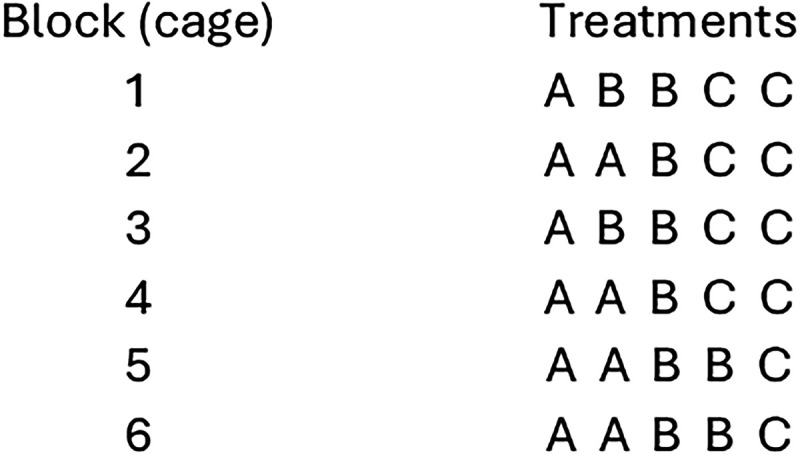
Random allocation plan for a balanced incomplete block design when treatments are fewer than block size. In this example there are three treatments (A, B, C) allocated to five mice (experimental units) in each cage (block factor).

Suppose the researcher wishes to study the effects of three treatments (A, B, C), with blocking on cage. The most efficient arrangement would be to house mice three to a cage and run the experiment as a randomized complete block design, with each treatment represented once in each cage. However, if housing density must be kept at the standard of five mice per cage, there will be fewer treatments than experimental units per cage. In this case, the design is in effect a complete block design with some treatments replicated within each block. Treatments are randomly allocated to each individually numbered mouse in each cage according to optimal design criteria. For example, for a single factor experiment with three treatments to be allocated to each cage of five mice is shown in Figure 6. An optimum random allocation plan generated with R *AlgDesign* (Extended Data 1, 6-1) balances treatments over the entire experiment: the three treatments are represented 10 times each and all treatment pairs are represented 16 times each.

#### Variable block sizes

Variation between litters is a major contributor to experimental noise and can directly affect the results of an experiment (Scott et al., 2008; Lazic and Essioux, 2013; Jiménez and Zylka, 2021; Warrington et al., 2025). However, the number of pups may vary considerably between litters, so blocking on litter can be problematic. Rather than balancing block size by culling extra pups, an alternative strategy that minimizes animal waste is to incorporate the natural variation in litter size directly in an incomplete blocked design. The disadvantage is that the total number of litters, litter sizes, and total number of pups must be known beforehand. However, with this information it is relatively straightforward to generate an optimal design and treatment allocation schedule with R *AlgDesign*. The algorithm distributes treatments across highly variable block sizes by maximizing D-optimality (or similar criteria). Treatment numbers are not necessarily balanced across blocks, but the distribution of treatments generated by the algorithm should maximize the precision of the treatment estimates, where precision is a measure of the efficiency of the design.

*Example*. A researcher wishes to investigate the effect of three drugs A, B, and C on growth of Swiss Webster mouse pups. The researcher can obtain data for 12 litters, totaling 107 pups. Because litter size and maternal environment affect pup growth, the researcher wishes to block on litter. However, litter size varies between 2 and 13 pups, so block size will be highly unbalanced.

**Figure 7.**
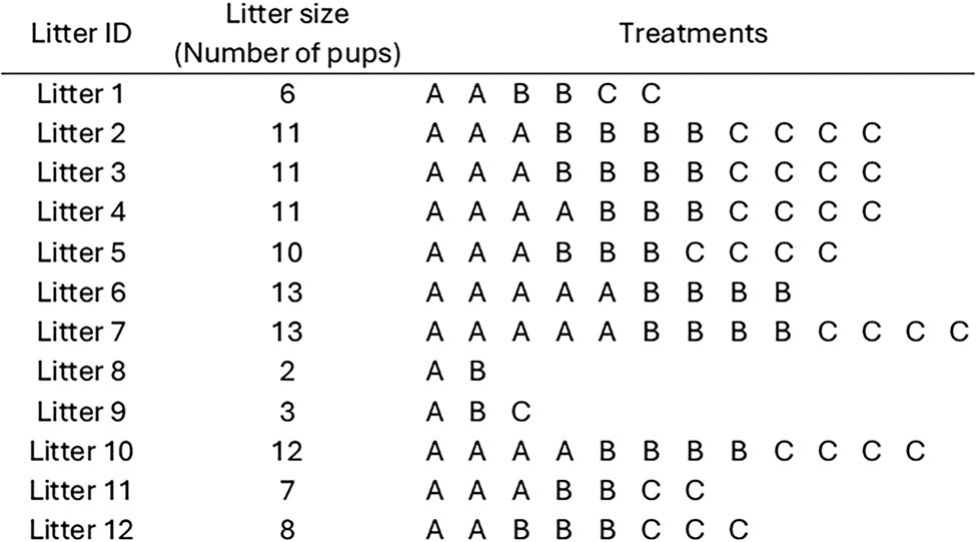
Random allocation plan for a blocked design with naturally variable block sizes. In this example there are three treatments (A, B, C) randomly allocated to 107 mouse pups (experimental units) distributed among 12 litters (blocks).

The random allocation plan generated in R *AlgDesign* optimizes the distribution of treatments across litters (Fig. 7; Extended Data 1, 7-1). The optimization algorithm produces the most precise estimates for treatment effects at the cost of slight imbalance in treatment sample sizes (*n_A_* = 36, *n_B_* = 35, *n_C_* = 32).

## Summary

“Block what you can and randomize what you cannot” (Box et al., 1978)

Blocking is an extremely efficient, flexible, and powerful method for minimizing the effects of unavoidable sources of variation in an experiment. However, if blocking is to be applied successfully, its incorporation in the study design requires careful planning ahead of time. The researcher must decide on a block factor most likely to account for the most unwanted variation in measurements of the outcome variables. Treatments must be allocated randomly with each block. This means that experimental units (usually animals) within each block must be individually identifiable to ensure the right treatments are applied to the right units. A coded identifier system improves study validity by assisting the researcher to minimize cognitive bias through allocation concealment and blinding (Day and Altman, 2000; Karp et al., 2022). Randomization minimizes systematic bias by distributing the effects of any remaining unknown or uncontrollable nuisance variables across treatment groups. Finally, “one size does not fit all”—the most appropriate block design for a given study must be decided upon first so that the random allocation plan can be customized to that design. Customization is essential to accommodate the specific block structure for that particular experiment, so that the error variance term used in later hypothesis tests will be correctly specified.

However, statistical control of unwanted variation will be ineffective unless the researcher controls the variation inherent to the experimental procedures themselves. Most unwanted variation results from inconsistent measurement processes and personnel competencies. Before the experiments begin, procedures should be standardized, and all technical staff trained to a uniform high standard. Animals should be physiologically stable before experimentation. This will involve implementation of low-stress, refined handling, housing, husbandry, and habituation procedures as part of regular standard of care (https://www.3hs-initiative.co.uk/additional-resources/protocols-sops-and-guidance-documents). Baseline stability of individual animals should be determined before data collection to ensure that the measured responses are due to the experimental conditions and not an artifact of the measurement setup. Habituation of research animals to test environments and testing apparatus is essential to minimize stress and novelty-induced anxiety that will affect behavioral and physiological measures (Saré et al., 2021). If animals are surgically instrumented, objective physiologically relevant measures such as heart and respiratory rates, blood pressure, lactate, and blood gases should be obtained and assessed, and any corrective measures implemented before data collection begins (Reynolds et al., 2019).

## Synthesis

Reviewing Editor: Mihaela Iordanova, Concordia University - Loyola Campus Decisions are customarily a result of the Reviewing Editor and the peer reviewers coming together and discussing their recommendations until a consensus is reached. When revisions are invited, a fact-based synthesis statement explaining their decision and outlining what is needed to prepare a revision will be listed below. The following reviewer(s) agreed to reveal their identity: Robert Calin-Jageman.

As editor for this series, I have already provided the author with a detailed review; this draft addresses all the comments I had provided.

I am especially enthusiastic about this 2nd part of the series. The first is a great primer on fundamentals; this second piece gives practical, workable advice on how to use blocking to isolate and measure the impact of potential confounds. It is clear, well organized, and chock full of excellent sources and examples.
