# Supplementary material for: Experimental Designs for Preclinical Neuroscience Experiments: Part 2—Blocking and Blocked Designs
Source: eNeuro. 2026 Feb 24;13(2):ENEURO.0006-26.2026. doi: 10.1523/ENEURO.0006-26.2026 (PMC12935487; doi:10.1523/ENEURO.0006-26.2026)
Supplement: Data 1 — Download Data 1, DOCX file. [file eneuro-13-ENEURO.0006-26.2026-s002.docx]

**Extended Data 1**

**1-1.** R code for random treatment allocation vs random sequence allocation

Random treatment allocation: Example for a single factor unblocked design with three treatments to be allocated to 24 mice (experimental units). Randomization is restricted to ensure a balanced design (sample size n = 8 per treatment)

#Set seed for reproducibility. Omit or change the seed #number to generate a new randomisation plan

set.seed(123)

#Define the total number of units

n_units <- 24

#Define the treatments

treatments <- c("A", "B", "C")

#Create a balanced allocation plan so that each treatment is represented 8 times in the design

assignment <- rep(treatments, length.out = n_units)

#Randomly shuffle treatments to remove ordering bias

random_order <- sample(assignment)

#Create the final data frame with mouse ID and Treatment

final_design <- data.frame(

UnitID = 1:n_units,

Treatment = random_order

)

# Print the assignment plan

print(final_design)

Random sequence order: Shuffle the order of 24 units

*#*list ID numbers

my_list <- 1:24

*#*randomly shuffle the list

shuffled_list <- sample(my_list, size = length(my_list), replace = FALSE)

*#Print the shuffled list*
print(shuffled_list)

*#*sample output

8 6 2 17 14 1 10 3 16 5 21 23 18 19 11 7 12 13 20 22 9 15 24 4

**2-1. Randomized complete block design**

Randomization plan for a randomized complete block design for four treatments A, B, C, D arranged in 6 blocks with 24 experimental units

#library(agricolae)

#Define the treatment levels

treatments <- c("A", "B", "C", "D")

#Define the number of blocks = replicates r

num_blocks <- 6

# Set seed for reproducibility

set.seed(123)

#'serie' determines the numbering of the experimental units within blocks (optional)

rcbd_design <- design.rcbd(trt = treatments, r = num_blocks, seed = 123)

#print the design

print(rcbd_design)

**2-2. Latin Square designs**

Randomization plan for a Latin Square design with four treatments and four levels for each block factor

#library(agricolae)
#Define the number of treatments (and therefore the number of rows and columns)

num_treatments <- 4

#Define the treatment names (e.g., A, B, C, D)
treatments <- LETTERS[1:num_treatments]

#Generate the Latin Square design
#The 'seed' argument ensures reproducibility. set.seed(123)
latin_square_design <- design.lsd(treatments, seed = 123)

#without seed for new allocation plan

latin_square_design <- design.lsd(treatments)

#print design
print(latin_square_design$sketch)

**Replicated Latin Square designs: Analysis**

**3-1**. Replicated 4 x 4 Latin Square with no nesting

#Analysis: replicate, row, and column factors are treated as independent blocks

model <- aov(Response ~ Rep + Row + Col + Trt, data = my_data)

#print results

summary(model)

**3-2**. Replicated 4 x 4 Latin Square with nesting of the row term in replicate

model <- aov(Response ~ Rep + Rep:Row + Col + Trt, data = my_data)

#print results

summary(model)

**3-3**. Replicated 4 x 4 Latin Square with nesting of both the row and column terms in replicate Rep

model <- aov(Response ~ Rep + Error(Rep/(Row*Col)) + Trt, data = my_data)

#print results

summary(model)

#alternatively the data can be analyzed as a nested mixed model

library(lme4)

library(lmerTest) #For p-values

# Nested structure: (1|Rep/Row) means Row is nested in #replicate Rep.

#Columns nested similarly, blocking factors are random #variables

model <- lmer(Response ~ Trt + (1|Rep) + (1|Rep:Row) + (1|Rep:Col), data = my_data)

#print results

anova(model)

**Incomplete block designs**

**5-1.**  **Randomization plan when number of treatments exceeds block size**

Example: Six treatments, five mice per cage, six cages. Values for

parameters *v*, *b*, *r*, *k*, and *λ* must be calculated before calling the program.

#library(ibd)

#enter parameter values(*v* = 6, *b* = 6,*r* = 5,*k* = 5,*λ* = 4)

bibd(6,6,5,5,4).

**6-1***.* **Randomization plan when block size exceeds number of treatment***s:* More experimental units than treatments in a block

Example: Three treatments, five mice per cage (“blocksizes”), six cages for a total of 30 mice (“nTrials”)

#library(AlgDesign)

cand <- data.frame(T=factor(c(rep("A", 10),rep("B", 10), rep("C", 10))) )

des <- optFederov(~ ., cand, nTrials=30)

des.blocked <- optBlock(~., des$design, blocksizes=c(5, 5, 5, 5, 5, 5))

des.blocked$Blocks

**7-1. Randomization plan when block size is naturally variable**

Example. Three treatments, twelve litters, with litters sizes 2, 3, 6, 7, 8, 10, 11, 11, 11, 12, 13, 13 (“blocksizes”)

#library(AlgDesign)

set.seed(123)

cand <- data.frame(T= factor(c(rep("A", 12), rep("B", 12), rep("C",12))) )

des <- optFederov(~ ., cand, nTrials=36)

des.blocked <- optBlock(~., des$design, blocksizes=c(6, 11, 11, 11, 10, 13, 13, 2, 3, 12, 7, 8))

des.blocked$Blocks
