# Supplementary material for: Experimental Designs for Preclinical Neuroscience Experiments: Part 2—Blocking and Blocked Designs
Source: eNeuro. 2026 Feb 24;13(2):ENEURO.0006-26.2026. doi: 10.1523/ENEURO.0006-26.2026 (PMC12935487; doi:10.1523/ENEURO.0006-26.2026)
Supplement: Data 2 — Download Data 2, DOCX file. [file eneuro-13-ENEURO.0006-26.2026-s003.docx]

**1-1**. Exemplary data describing average partial pressure of brain tissue oxygen (BrPO_2_, mmHg) for 24 mice in six cages (blocks). Treatments are randomly allocated to mice in each cage, and all four treatments are represented in each block.

| Block=cage | Treatment | BrPO_2_ |  | Block=cage | Treatment | BrPO_2_ |
| --- | --- | --- | --- | --- | --- | --- |
| 1 | C | 16.8 |  | 4 | B | 20.3 |
| 1 | A | 15.5 |  | 4 | A | 20.7 |
| 1 | D | 17.2 |  | 4 | D | 22.9 |
| 1 | B | 16.4 |  | 4 | C | 20.7 |
| 2 | A | 17.7 |  | 5 | A | 20.6 |
| 2 | B | 17.6 |  | 5 | D | 23.7 |
| 2 | D | 19.8 |  | 5 | B | 22.2 |
| 2 | C | 18.5 |  | 5 | C | 21.4 |
| 3 | D | 19.8 |  | 6 | C | 23.3 |
| 3 | B | 18.5 |  | 6 | D | 23.9 |
| 3 | C | 19.9 |  | 6 | B | 22.2 |
| 3 | A | 19.7 |  | 6 | A | 23.1 |
